# Extraction of Lycopene from Tomato Peels Using Supercritical Carbon Dioxide and Conjugation of the Extracted Lycopene with TiO_2_ Nanoparticles

**DOI:** 10.1021/acsomega.5c11461

**Published:** 2026-02-23

**Authors:** Farid Hajareh Haghighi, Roya Binaymotlagh, Lionel Nguemna Tayou, Marianna Villano, Laura Chronopoulou, Cleofe Palocci

**Affiliations:** † Department of Chemistry, 9311 Sapienza University of Rome, Piazzale Aldo Moro 5, Rome 00185, Italy; ‡ Research Center for Applied Sciences to the Safeguard of Environment and Cultural Heritage (CIABC), Sapienza University of Rome, Piazzale Aldo Moro 5, Rome 00185, Italy

## Abstract

The tomato processing industry is one of the most widespread food manufacturing sectors globally, generating substantial amounts of residue, including tomato skins, peels, seeds, and vascular tissues. These residues still retain valuable bioactive compounds (e.g., carotenoids like lycopene), essential for food, pharmaceutical, and nutraceutical applications. Currently, traditional solvent extraction is the most common method for retrieving these compounds from tomato residue. However, this approach has notable disadvantages, including high solvent consumption and difficulties in utilizing leftover biomass. To address these issues, innovative technologies have introduced modifications to process configurations and techniques that alter or break down plant cells, significantly improving compound recovery. Supercritical fluid extraction offers an effective method for enhancing the value of tomato residues before disposal, as it is an environmentally friendly approach, particularly when carbon dioxide is used as the extraction solvent. The novelty of the present work is the specific optimization and integration of this green extraction technique with the subsequent conjugation of the extracted lycopene to TiO_2_ nanoparticles (TiO_2_NPs). This is the first report demonstrating the conjugation of supercritical CO_2_-extracted lycopene with TiO_2_NPs, effectively eliminating the use of organic-solvent-derived carotenoids traditionally used in such conjugates. The supercritical CO_2_-extraction (scCO_2_) was employed at various times (2, 3, and 4 h), temperatures (40–80 °C), and pressures (25, 30, and 35 MPa). Among the tested protocols, the treatment of tomato at 50 °C and 30 MPa for 2 h showed the highest yield based on the UV–vis and HPLC results. For the potential pharmaceutical benefits of these extracts, lycopene was directly conjugated with TiO_2_NPs, using a mild and green approach (TiO_2_NPs-lycopene) and characterized using different techniques, including HPLC, UV–vis, FTIR-ATR, FESEM-EDS, and DLS to assess the successful loading of 95.0 ± 2.1% of lycopene on the TiO_2_NPs. The FESEM results exhibited the grain-like TiO_2_NPs-lycopene particles with a 60–80 nm size distribution, indicating size-controlling effect of the lycopene on the TiO_2_NPs during the conjugation process and the presence of the organic molecule layer on the surface. TiO_2_NPs-lycopene represents a promising candidate for antimicrobial studies due to the potential synergic effect of TiO_2_NPs and lycopene in a single nanoplatform. The results show the importance of scCO_2_-based techniques, as valid alternatives to the conventional methods that exploit organic solvents, to prepare the TiO_2_NPs-carotenoids conjugates. To the best of our knowledge, this is the first study to report the conjugation of TiO_2_NPs with lycopene, offering a novel approach to enhance their antioxidant and photoprotective properties.

## Graphical abstract

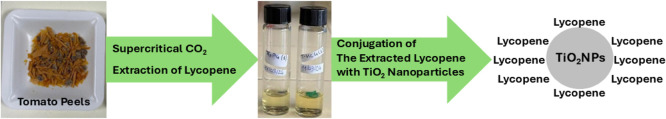

## 1 Introduction

Tomatoes play a crucial role in human diets worldwide and they are linked to various health benefits, primarily due to their carotenoid content, especially lycopene.^1,2^ This powerful antioxidant helps protect against cardiovascular diseases and other health conditions.^3,4^ While some tomatoes are consumed fresh, most are processed into various products, and their production continues to rise.^5,6^ However, this growing industry also generates significant waste—pomace, seeds, peels, skins, juices, and pastes—which can account for up to 40% of the raw material.^7,8^ Although much of this waste is either discarded on land or repurposed for animal feed,^9,10^ its rich composition could present opportunities for recovering nutrients and bioactive compounds (e.g., lycopene and β-carotene) for applications in food, pharmaceuticals, and nutraceuticals, as a promising opportunity-enhancing sustainability, benefiting the industry, and providing advantages for consumers while reducing environmental impact.^11,12^

High-value compounds in plants are often trapped within cell structures, creating a barrier that limits their extraction through conventional solvent-based methods.^13,14^ Traditionally, vegetable material extraction relies on organic solvents, but their harmful effects on human health and the environment pose significant challenges.^15,16^ This is especially problematic for industries such as food, pharmaceuticals, and cosmetics, as well as from an economic standpoint due to stringent regulations and costly waste management requirements.^17,18^ Green chemistry principles emphasize minimizing waste and reducing reliance on hazardous solvents and chemicals to promote safer, more sustainable, and energy-efficient processes.^19,20^

In line with these principles, research has explored the potential of more environmentally- and eco-friendly processes and solvents,^21,22^ including microwaves,^23,24^ pulsed electric fields,^25,26^ ultrasounds,^27,28^ pressurized solvents,^29,30^ green solvents,^31,32^ enzymatic pretreatments,^33,34^ and biosolvents.^35^ Supercritical fluid extraction, particularly when using CO_2_ (as it serves as a versatile solvent), can be adjusted based on the properties of the target compounds while allowing for environmentally safe separation of the extracted solutes.^36–38^ Although supercritical ethane has been explored, supercritical CO_2_ (scCO_2_) remains the preferred option due to its low critical point (73.8 bar and 31.0 °C), which facilitates the extraction of heat-sensitive compounds like carotenoids.^39,40^ Its widespread availability, affordability, nontoxic nature, and safety further contribute to its advantages.^41,42^ Adjusting pressure and temperature significantly influences the density, viscosity, diffusivity, and solubility of the supercritical solvent, improving penetration into porous matrices and enhancing mass transfer.^43,44^ Additionally, fine-tuning the solvating ability of scCO_2_ is crucial for optimizing extraction and effectively separating extracts through depressurization.^45^

Lycopene possesses extensive conjugated double bonds (Figure S1), which make it effective antioxidants—but also chemically unstable under ambient conditions.^46,47^ At room temperature, it is particularly susceptible to oxidation, isomerization, and degradation when exposed to light, oxygen, and heat.^48,49^ Lycopene tends to degrade faster than β-carotene due to its more linear structure and, more importantly, its higher degree of unsaturation, which makes it more prone to isomerization from the all *trans* form to less bioactive *cis*-isomers.^50,51^ Both compounds can undergo photo-oxidation, leading to a loss of color and antioxidant activity.^52^ To preserve their stability, they are often stored in dark, airtight containers and sometimes encapsulated using nanocarriers like liposomes or solid lipid nanoparticles.^53^

Conjugating or entrapping carotenoids with nanoparticles is a promising strategy to enhance their chemical stability, especially against degradation caused by light, oxygen, and heat.^54^ When bound to nanoparticles, these pigments benefit from the protective matrix and photostability of the nanocarrier, which can shield them from environmental stressors and reduce isomerization and oxidation rates.^55^ Moreover, the nanoparticle’s surface can be modified to improve compatibility with lipophilic carotenoids, allowing for better dispersion and stronger interactions.^56^ This not only stabilizes the carotenoids but also enhances their bioavailability and controlled release, making the conjugates more effective in biomedical and nutraceutical applications. Some studies even suggest that carotenoids can act as capping agents, further improving the stability and functionality of the nanoparticles themselves.^57^

Titanium dioxide (IV) nanoparticles (TiO_2_NPs) have emerged as a cornerstone in biological science due to their unique physicochemical properties, including high surface area, photocatalytic activity, and biocompatibility.^58^ These features make them highly effective in a range of biomedical applications such as drug delivery,^59^ antimicrobial treatments,^60,61^ cancer therapy,^62,63^ biosensing,^64^ and tissue engineering.^65,66^ Their ability to generate reactive oxygen species under light exposure is particularly valuable in photodynamic therapy, where they can selectively destroy cancer cells or pathogens.^63,67^ TiO_2_NPs also find widespread application in the cosmetic industry, which is commonly incorporated into sunscreen products as an inorganic UV-blocking agent. Its ability to form optically transparent films on the skin makes it ideal for such uses. TiO_2_ exists in three crystalline phases—anatase, brookite, and rutile—with the rutile form being preferred in sunscreens due to its superior UV absorption capacity.^68^ To further enhance their UV-blocking efficiency, TiO_2_NPs can be surface-coated, enabling improved light diffraction mechanisms. Beyond sunscreens, TiO_2_NPs are also utilized in various personal care products such as antiwrinkle creams, lip balms, and toothpaste.^69^

Conjugation of carotenoids to TiO_2_NPs enhances their stability and bioavailability, overcoming the limitations of poor solubility and rapid degradation in physiological environments. The antioxidant properties of carotenoids, when coupled with the photocatalytic and drug-carrying capabilities of TiO_2_NPs, create a synergistic platform for oxidative stress-related disease therapies.^63,64^ TiO_2_NP-carotenoid conjugates enable targeted delivery and controlled release of therapeutic agents, minimizing systemic toxicity and improving treatment efficacy.^60,70^ The hybrid system offers dual functionality—protective antioxidant action from carotenoids and diagnostic or therapeutic utility from TiO_2_NPs—making it ideal for theranostic applications. Conjugation improves cellular uptake and retention of carotenoids, allowing for more effective intervention in diseases such as cancer, neurodegeneration, and cardiovascular disorders.^63,64^

In our study, the CO_2_-supercritical extraction (scCO_2_) was employed to extract lycopene from the tomato peels employing different times (2, 3, and 4 h), temperatures (40–80 °C), and pressures (25, 30, and 35 MPa) for the scCO_2_ treatment. Subsequently, the conjugation of the extracted lycopene with TiO_2_NPs was studied using a mild and green approach, and then they have been physicochemically characterized using different techniques, including HPLC, UV–vis, FTIR-ATR, FESEM-EDS, and DLS. The combination of some carotenoids with TiO_2_NPs has been revealed in previous studies;^71,72^ however, their conjugation with lycopene has not been previously reported.

This study is the first to investigate this new hybridization. Lycopene offers superior biological benefits when conjugated with TiO_2_ compared to other carotenoids due to its potent antioxidant activity, cancer-preventive properties, and enhanced cellular uptake, making it especially valuable for potential biomedical applications such as drug delivery, photodynamic therapy, and tissue protection.^73,74^

## 2 Materials and Methods

### 2.1 Materials

Tomato peels (TP), the agro-industrial byproduct herein used, were provided by an Italian company involved in TP valorization (with a moisture content of approximately 6.1 ± 1.1 wt %). Analytical- and HPLC-grade chemicals, organic solvents, commercially available titanium (IV) oxide nanopowder (TiO_2_NPs) (*d*_ave_ ∼ 25 nm, anatase >97%), pure lycopene, and β-carotene (to prepare the standard solutions for the calibration curves) were purchased from Aldrich Chemical.

### 2.2 Instruments

Ultrapure water (resistivity of 18.3 MΩ·cm) was prepared using a Zeneer Power I Scholar-UV deionizer (Full Tech Instruments). The TiO_2_NPs-carotenoids colloids were purified via centrifugation at 13400 rpm for 20 min at 8 °C using a Scilogex centrifuge, followed by filtration through a 0.22  μm membrane. The carotenoid content was quantified using a Waters 1525 HPLC system with a Dual λ Detector (Waters 2487) and a Symmetry C18 column (4.6 × 100 mm, 5  μm). The flow rate was set to 2 mL/min at a pressure of 70 bar; the mobile phase of methanol: acetonitrile in a 9:1 v/v ratio (containing 0.125% of triethylamine) was used. The UV–vis detector was set at 475 nm, and approximate retention times of around 6.5 and 8.5 min were obtained for lycopene and β-carotene, respectively. Both molecules are nonpolar hydrocarbons, but lycopene is considered slightly more polar than β-carotene due to its extended conjugation and electron distribution. Lycopene elutes earlier than β-carotene due to its linear structure and extensive electron delocalization, which result in slightly weaker interactions with the stationary phase (Figure S1).^75,76^ The calibration curve was obtained by analyzing pure standard solutions of the carotenoids (0.1–10  ppm) (Figures S2 and S3). UV–vis spectra were measured using a Varian Cary 100 spectrophotometer across 200–800 nm at ambient temperature with quartz cuvettes (1  cm path length). Particle size (⟨2*R*_H_⟩, nm) and zeta potential were assessed through dynamic light scattering (DLS) using a Malvern Zetasizer Nano-ZS90 with a 633  nm He–Ne laser at 25 °C. All size and stability tests were performed in triplicate and reported as mean ± standard deviation. FT-ATR spectra were obtained using a Bruker Vertex 70 in ATR mode (4000–600  cm^–1^, 32 scans, 4  cm^–1^ resolution). Nanohybrid morphology was examined with FESEM (Auriga Zeiss) supported by an EDS detector. Samples were drop-cast onto conductive silicon. The scCO_2_ tests were performed in triplicate with duplicate samples. The results were expressed in terms of average values ± standard deviations. Statistical analysis of the data was performed by univariate analysis of variance (ANOVA) with a significance level of 95% (*p* < 0.05) using the Tukey’s test.

### 2.3 Grinding and Homogenization of Tomato Peels

The freeze-dried granules of tomato peels were grinded with a ceramic mortar and pestle, followed by a mechanical mixer, to reduce their particle size and increase contact between the particles and the scCO_2_, facilitating scCO_2_ entry into the cell (Figure S4).

### 2.4 Solvent Treatment of Tomato Peels

The extraction with solvent was carried out with an amount of 2.5 g of tomato peels in 100 mL of a solution of hexane/acetone/ethanol in a ratio of 2:1:1 v/v. Once prepared, the mixture was stirred for 3 h at room temperature. Once the extraction was completed, the supernatant was collected using Pasteur pipettes and placed in 5 mL Eppendorf tubes for purification using a centrifuge set at a speed of 5000 rpm for 10 min at 25 °C. Finally, the supernatant was removed from each Eppendorf to obtain solutions free of solid residues on which further analyses could be conducted.

### 2.5 scCO_2_ Treatment of Tomato Peels

The extraction process was performed in a stainless-steel scCO_2_ tubular reactor with a volume of 1 cm^3^, where the solid samples were placed. The liquid CO_2(l)_ was pumped into the high-pressure reactor, then pressurized to a specific target pressure using a syringe pump and heated to the set temperature via a circulating air system within a thermostat-controlled chamber housing the reactor (Figures S5 and S6). Each experiment included an initial static extraction, followed by a 10 min dynamic extraction step, during which supercritical CO_2_ was depressurized to wash the extracted carotenoids into 3 mL of ethanol (Figure S7). For each extraction test, a fixed amount of dried tomato peels (230 mg) was placed in the cell without addition of cosolvents. In this study, the operating parameters varied in the pressure range of 20–30 MPa, temperature of 40–80 °C, and time of 2–6 h. The ethanolic solutions containing the extracts were used for quantitative evaluation of the extracted lycopene and β-carotene.

### 2.6 Immobilization Procedure

To determine the optimal carotenoid loading, various weights of TiO_2_NPs (ranging from 1–5 mg TiO_2_NPs) were tested, and Table S1 only shows the five selected reaction conditions for the tests. In each case, the same conjugation protocol was employed: the TiO_2_NPs were added directly to extracted lycopene (initial concentration of 1.8 ppm), resulting in a colloidal mixture. The suspension was sonicated for 15 s in a 50 MHz ultrasonic bath, followed by continuous magnetic stirring at room temperature for various times (3–48 h), protected from light. All of the loading tests were performed in quadruplicate (Figure S8). After the conjugation step, TiO_2_NPs-carotenoids complexes were isolated using centrifugation (13400 rpm, 8 °C, 20 min) and washed three times with ultrapure water to remove unbound molecules. The final conjugates were freeze-dried and stored at 4 °C for further use.

## 3 Results and Discussion

### 3.1 scCO_2_-Based Extraction of the Carotenoids at Different Temperatures

Extractions were first performed by varying oven temperatures (40, 50, 60, 70, and 80  °C) while maintaining a constant CO_2_ pressure of 30 MPa. Each experiment involved a static extraction phase of 2 h, followed by a dynamic extraction step lasting 10 min to collect the extracted carotenoids. The resulting extracts were subsequently analyzed by using UV–vis spectrophotometry to compare the results. Based on their UV–vis intensities, the highest extraction yield was achieved at 50 °C (Figures 1 and S9).

**Figure 1:**
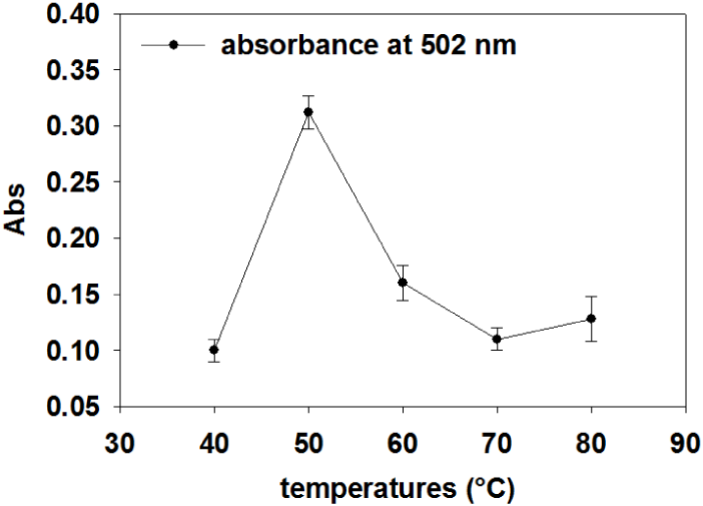
UV–vis absorption at 502 nm of fresh scCO_2_-extracted samples at different temperatures (at 30 MPa for 2 h).

Beyond this temperature, a decline in absorbance was observed, suggesting the thermal degradation of carotenoids. Both lycopene and β-carotene absorb strongly in the 450–500 nm range. In nonpolar solvents, they typically show three major absorption peaks in the visible spectrum at ∼445, 470, and 502 nm for lycopene, and ∼427, 450, and 466 nm for β-carotene. These peaks correspond to π → π* electronic transitions in the conjugated polyene chain (see Figures S1 and S9).

The spectral overlapping of lycopene and β-carotene is a well-documented challenge in spectroscopic analysis due to their similar molecular structures and absorption characteristics.^77^ UV–vis spectroscopy alone can provide qualitative insights and rough quantification of lycopene and β-carotene, especially when absorbance ratios or derivative methods are employed. However, for precise discrimination, especially in mixed or complex samples, it is best paired with HPLC. The HPLC spectrum of this extract clearly shows the presence of lycopene and β-carotene.^78^

Temperature plays a vital role in the characteristics of scCO_2_ and its efficiency. Based on the literature, most of the studies were conducted at temperatures ranging from 40 to 80  °C—a range commonly used for extracting compounds from plant-based materials.^79^ Much like pressure, temperature has a major influence on the density of scCO_2_, especially at lower pressures.^80^

As mentioned in the introduction section, both lycopene and β-carotene degrade over time primarily due to oxidation and *trans*-to-*cis* isomerization, processes that are accelerated by environmental factors like heat, light, and oxygen.^81^ On this basis, after the extractions at different temperatures, the samples were stored at 4 °C and their UV–vis spectrum was monitored over 30 days (Figure 2). The results show a decrease in the absorbance over time, which might be due to the degradation process of the stored samples.

**Figure 2:**
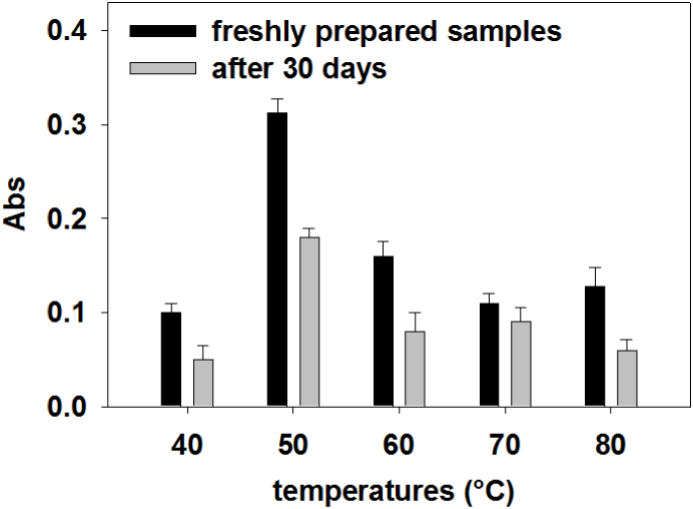
UV–vis stability study for the scCO_2_ extractions performed at different temperatures after 30 days.

### 3.2 scCO_2_-Based Extraction of the Carotenoids at Three Different Pressures

By keeping constant the extraction time (2 h) and oven temperature (50  °C), the extractions were performed at three different pressures of 25, 30, and 35 MPa. Figure 3 shows the UV–vis absorbances of the extracted samples demonstrating the maximum absorption for the 30 MPa test. It is well established that increasing pressure enhances the density and solvating ability of the solvent, which can boost the solubility of the solute and accelerate the extraction process. Additionally, operating at elevated pressures may reduce the amount of CO_2_ required to achieve similar extraction yields.^82^ However, higher pressures can also lead to decreased solvent diffusivity. Excessive pressure may compress the sample matrix, reduce pore size, and increase packing density, which can negatively impact extraction efficiency.^83,84^ Combining pressure with optimal temperature and CO_2_ flow rate is key to maximizing yield and preserving carotenoid’s antioxidant properties.

**Figure 3:**
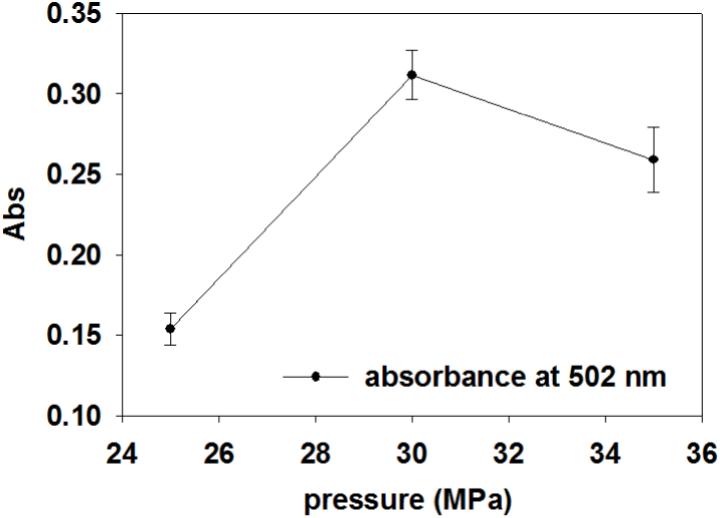
Absorbances (at 502 nm) of the scCO_2_ extractions at 25, 30, and 35 MPa.

### 3.3 scCO_2_-Based Extraction of the Carotenoids at Three Different Times

At constant pressure (30 MPa) and temperature (50  °C), the scCO_2_ extractions were conducted at three different extraction times: 2, 4, and 6 h, in which the best results were obtained for 2 h (Figures 4 and S10). Extraction time significantly influences the efficiency and yield of carotenoids during scCO_2_ extraction. Extended extraction times can improve yield but may also 1) increase energy and CO_2_ consumption; and 2) increase the risk of thermal degradation of carotenoids if temperature is not well controlled, therefore optimizing time is crucial for balancing efficiency and cost-effectiveness.^85^

**Figure 4:**
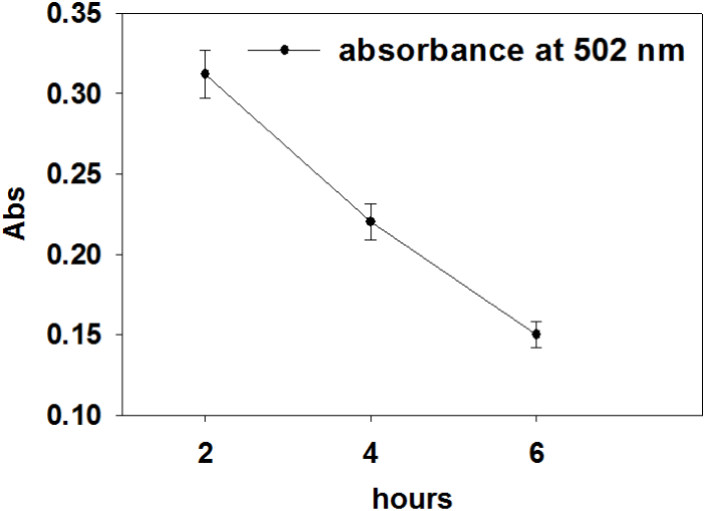
UV–vis absorption (at 502 nm) of scCO_2_-extracted samples at various times (at 30 MPa and 50  °C).

### 3.4 Comparison of Solvent and scCO_2_ Extractions in Terms of Selectivity and Extraction Efficiency

The HPLC results of the solvent-based extraction were compared with the best scCO_2_ extraction test (at 50 °C, 30 MPa, and 2 h) (Table 1).

**Table 1:** Selectivity and Quantity (in ppm) of the Carotenoids Extracted in Solvent and scCO_2_ Methods

the extraction method	lycopene (ppm)	β-carotene (ppm)
solvent	0.24 ± 0.01	0.54 ± 0.01
scCO_2_	0.14 ± 0.02	0.75 ± 0.03

The results indicate that lycopene extraction using the solvent method is twice as high as that obtained with scCO_2_ (Figures S11 and S12). Conversely, the β-carotene content extracted by scCO_2_ is 1.5 times higher compared with the solvent-based method. Interestingly, the scCO_2_ extraction also yielded lutein at around 5.8 min, as seen in Figure S12.

### 3.5 Conjugation of the Extracted Lycopene with TiO_2_NPs

For the first time, the direct conjugation of extracted lycopene onto the surface of TiO_2_NPs nanoparticles was investigated, paving the way for future work on potential applications in dermatological therapies, antioxidant delivery systems, and photoprotective formulations for skin-related medical conditions, which is mentioned in more detail in Section 1. The procedure was carried out under mild conditions, as shown in Figure S8, using ethanol as the medium. The nanoparticles were allowed to interact with the carotenoids while being stirred at room temperature in the absence of light.

Experiments were conducted at different TiO_2_NPs weights (1–5 mg) and reaction times 3–48 h (five selected conditions are shown in Table S1). UV–vis spectroscopy and HPLC/UV–vis were employed to gain valuable information for quantifying the amount of conjugation following its interaction with the TiO_2_NPs. The loading of carotenoids was determined using established equations^86^ to calculate the drug loading efficiency (η%), eq 1:

1 $${\mathrm{Loading}\;\mathrm{efficiency},\;\eta \%=W}_{\mathrm{loaded}\;\mathrm{drug}}{/W}_{\mathrm{free}\;\mathrm{drug}}\times \mathrm{100}$$

where *W*_loaded lycopene_ and *W*_free lycopene_ represent the weights of the loaded drug and unbound (free) drug, respectively.

Quantitative analysis was performed using the calibration curve obtained via HPLC (Figures S2 and S3), based on known concentrations of lycopene. The best lycopene loading was obtained with 1.4 mg of TiO_2_NPs for 24 h (Table S1).

Figure 5 displays the HPLC chromatograms of the initial lycopene loading solution (1.8 ppm) and the supernatant collected after 24 h of interaction with TiO_2_NPs.

**Figure 5:**
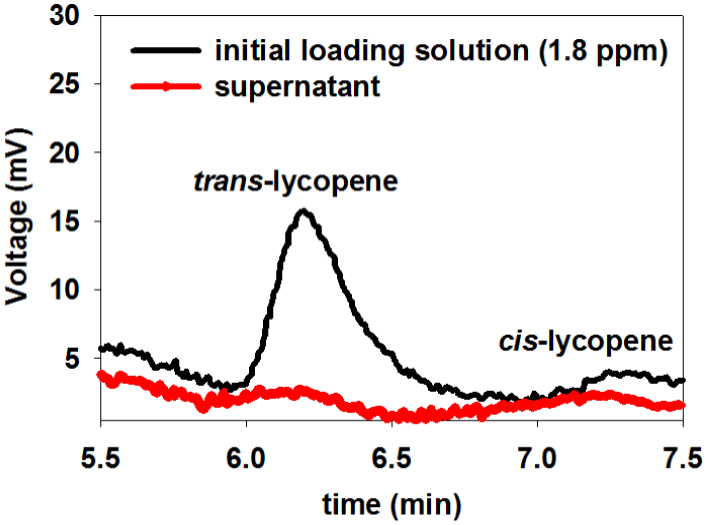
HPLC chromatogram of lycopene loading solution, compared with supernatant suspension after interaction (for 1.4 mg of TiO_2_NPs, 24 h reaction time, 1.8 ppm for initial concentration of lycopene).

A noticeable reduction in the absorption peak intensity of the supernatant, compared with the original loading solution, indicates successful lycopene conjugation onto the nanoparticle surface. By comparing the concentrations of the two solutions using the HPLC calibration curve (Figure S2), a 95.0 ± 2.1% loading capacity (34.2 μg loaded lycopene) was obtained for this reaction condition.

The conjugation mechanism of lycopene onto the surface of TiO_2_NPs in an organic solvent involves both chemical interactions and physical adsorption, and can be influenced by the solvent environment, the surface chemistry of TiO_2_, and the structure of lycopene. Proposed conjugation mechanisms could include the following:

1. π*–*π Interactions and surface adsorption: Lycopene, a highly conjugated polyene, can adsorb onto the TiO_2_ surface through π*–*π stacking and van der Waals forces. In organic solvents like ethanol, these interactions are stabilized due to reduced polarity compared to aqueous systems.
2. Ligand-like coordination: Lycopene may act as a ligand, forming a complex with surface titanium atoms. One study suggests that C=C double bonds in lycopene can interact with Ti­(IV) centers, while oxygen atoms (if present in oxidized lycopene) may coordinate with surface hydroxyl groups on TiO_2_NPs. As supporting evidence, a report proposed that lycopene can serve as both a reducing agent and a ligand, forming a lycopene-TiO_2_ complex through direct interaction between titanium ions and the polyene chain.
3. Solvent-mediated stabilization: Organic solvents help disperse lycopene and prevent aggregation, allowing better access to the TiO_2_ surface. Solvents also influence the orientation and conformation of lycopene during binding.

ATR spectra of TiO_2_NPs-lycopene were recorded after 30 days (Figure 6), with the assignment of their main characteristic bands in the 4000–600 cm^–1^ range. In this spectrum, all the spectral features are seen including the following: 957 cm^–1^ (the most prominent and diagnostic peak, corresponding to the *trans* C–H deformation vibration of the polyene chain in lycopene^87^); 1150–1250 cm^–1^ (C–C stretching vibrations); ∼1370–1450 cm^–1^ (CH_2_ and CH_3_ bending vibrations); and ∼1650 cm^–1^ (C=C stretching of the conjugated double bonds confirms the presence of lycopene on the TiO_2_ nanosurface).

**Figure 6:**
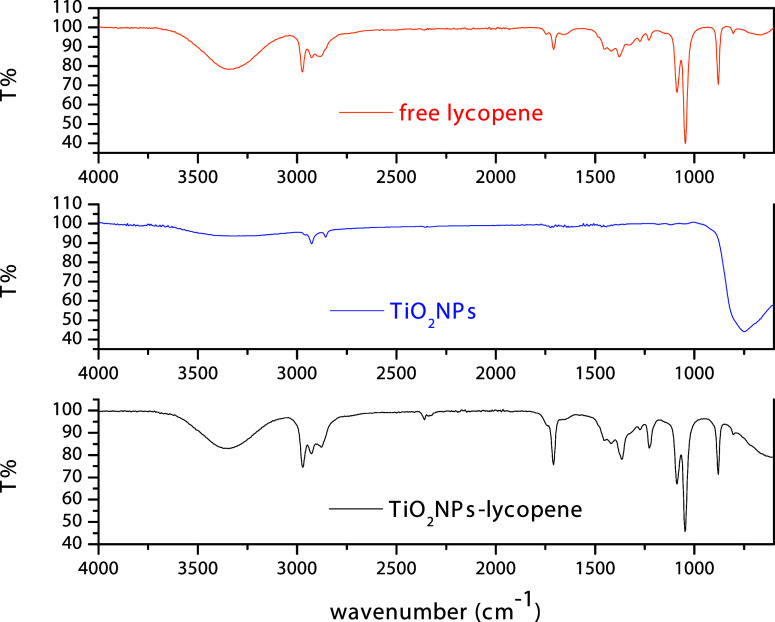
ATR spectra of TiO_2_NPs-lycopene, TiO_2_NPs, and lycopene.

Morphology and size of TiO_2_NPs and TiO_2_NPs-lycopene were evaluated using the FESEM technique. Comparing the pristine and loaded TiO_2_NPs (Figure 7), a different pattern can be observed in FESEM images in terms of shape and size distribution. The TiO_2_NPs-lycopene nanoconjugates are grain-like. Concerning the size of TiO_2_NPs-lycopene, the FESEM micrographs show a size distribution of 60–80 nm, which means that the conjugation process protects nanoparticles from aggregation. This phenomenon could be attributed to the presence of lycopene on the TiO_2_NPs, which prevents their size growth and overall has a stabilizing effect on the particles.

**Figure 7:**
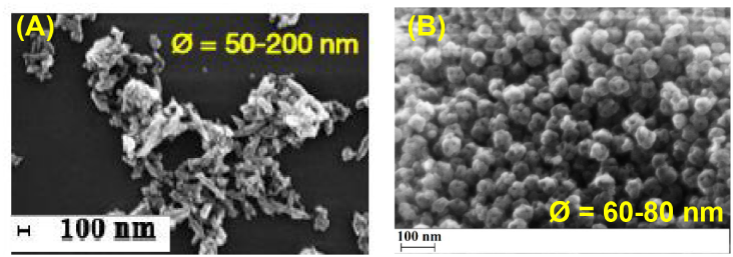
SEM of TiO_2_NPs before (A) and after (B) the conjugation.

To assess colloidal behavior, the hydrodynamic diameter and ζ-potential of the suspensions were measured (at neutral pH) to investigate the surface characteristics and stability of TiO_2_NPs and TiO_2_NPs-lycopene and, more importantly, the changes in these two properties upon lycopene conjugation. For the bare TiO_2_NPs, the results showed a hydrodynamic diameter of (295 ± 200) nm and a ζ-potential of (−15 ± 4) mV, demonstrating their moderate stability in the solution. These results are consistent with the literature, which reported that TiO_2_NPs are stable only in the low pH (<2.0) and high pH (>9.0) regions, and the smallest NP aggregation is reported.^88^ For this reason, the DLS study was performed at neutral pH condition (7.0–7.5), which is near the pH_PZC_ of TiO_2_NPs (PZC: point of zero charge); in this condition, the surface hydroxyl groups of TiO_2_NPs do not provide high stability for the colloidal suspension. Conjugation of lycopene results in increasing the surface charge to (−22 ± 5) mV and decreasing the hydrodynamic size to 240 ± 110 nm, confirming the change in surface characteristics of nanoparticles, compared to the TiO_2_NPs alone. The increase in ζ-potential can be attributed to the presence of the lycopene molecules on the surface of TiO_2_NPs. The colloidal stability of TiO_2_NPs-lycopene was monitored over 4 h by recording their UV–vis spectra. As can be seen in Figure 8, TiO_2_NPs-lycopene remain 80% of their colloidal stability after 4 h in H_2_O.

**Figure 8:**
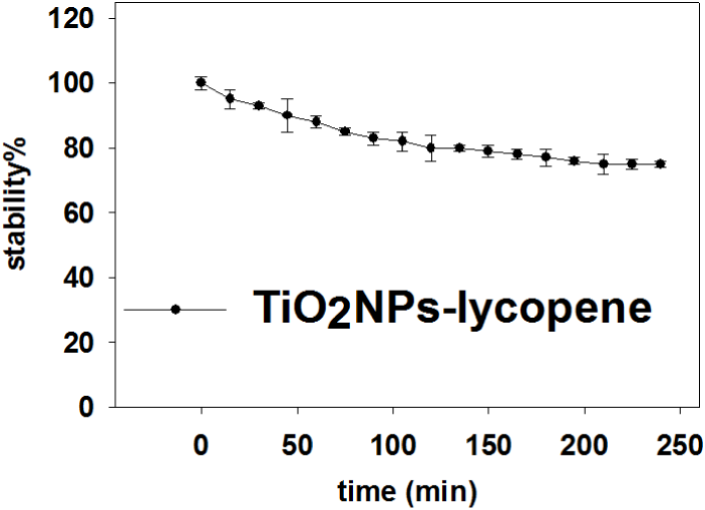
Stability test for TiO_2_NPs-lycopene.

## 4 Conclusion

This study demonstrated the extraction of lycopene and β-carotene from tomato peels based on the scCO_2_ extraction process. The effects of time, pressure, and temperature were investigated. The yield of carotenoids obtained using scCO_2_ extraction is comparable to that achieved through conventional solvent extraction methods. Furthermore, by optimization of the extraction parameters of the scCO_2_ process, the selectivity of carotenoid extraction can be modulated. For the first time, TiO_2_NPs-lycopene conjugates were prepared, optimized, and analyzed using a range of characterization techniques. The results demonstrated that lycopene can be effectively loaded onto photoactive TiO_2_NPs using an environmentally friendly method. HPLC analysis confirmed successful loading with an efficiency of η = 95.0 ± 2.1%. The encouraging results have stimulated our research toward future biomedical applications of such bioconjugates. In fact, further steps will focus on studying the synergic effect of lycopene with TiO_2_NPs, evaluating their antioxidant and antimicrobial activities against various pathogenic microorganisms. This study demonstrates, for the first time, the successful conjugation of TiO_2_NPs with lycopene, establishing a promising foundation for further studies of multifunctional cosmetic and therapeutic formulations. The synergistic enhancement of UV protection and antioxidant activity opens new avenues for future research and product development.

## Supplementary Material



## Notes

Chemical structure of lycopene and β-carotene; calibration curve of lycopene in the concentration range of 0.1–10 ppm; calibration curve of β-carotene in the concentration range of 0.1–10 ppm; the freeze-dried granules of tomato peels before (A) and after (B) grinding; the scheme of the extraction system; (A) side view (internal) part of the scCO_2_ instrument and (B) its processing keyboard; collecting carotenoids from the cell into 3 mL of ethanol, after the scCO_2_ extraction; conjugation of TiO_2_NPs with pure lycopene (or β-carotene); UV–vis spectrum of the scCO_2_ extracted sample at 50 °C, 30 MPa, 2 h (inset: the photo of the extracted samples); UV–vis spectrum of scCO_2_ extracted sample at 50 °C, 30 MPa, for different times; HPLC chromatogram of the solvent extraction test; HPLC chromatogram of the best scCO_2_ extraction test (at 50 °C, 30 MPa, and 2 h); the five selected reaction conditions for the TiO_2_NPs-lycopene conjugation (PDF)
