# Supplementary material for: Extraction of Lycopene from Tomato Peels Using Supercritical Carbon Dioxide and Conjugation of the Extracted Lycopene with TiO2 Nanoparticles
Source: ACS Omega. 2026 Feb 23;11(9):14919–28. doi: 10.1021/acsomega.5c11461 (PMC12980429; doi:10.1021/acsomega.5c11461)
Supplement: Supplementary file 1 [file ao5c11461_si_001.pdf]

Supporting Information (SI) of

Extraction of Lycopene From Tomato Peels Using Supercritical Carbon Dioxide and Conjugation of The Extracted Lycopene with TiO_2 Nanoparticles

Farid Hajareh Haghighi[a], Roya Binaymotlagh[a], Lionel Nguemna Tayou[a], Marianna Villano[a,b], Laura Chronopoulou[a,b*], Cleofe Palocci[a,b*]

[a]Department of Chemistry, Sapienza University of Rome, Piazzale Aldo Moro 5, 00185 Rome, Italy

[b]Research Center for Applied Sciences to the Safeguard of Environment and Cultural Heritage

(CIABC), Sapienza University of Rome, Piazzale Aldo Moro 5, 00185 Rome, Italy

Number of Pages: 8

Number of Figures: 12

Number of Tables: 1

***Corresponding authors,**

Cleofe Palocci and Laura Chronopoulou, e-mail addresses: cleofe.palocci@uniroma1.it, laura.chronopoulou@uniroma1.it, Phone number: 0039-06 4991 3317

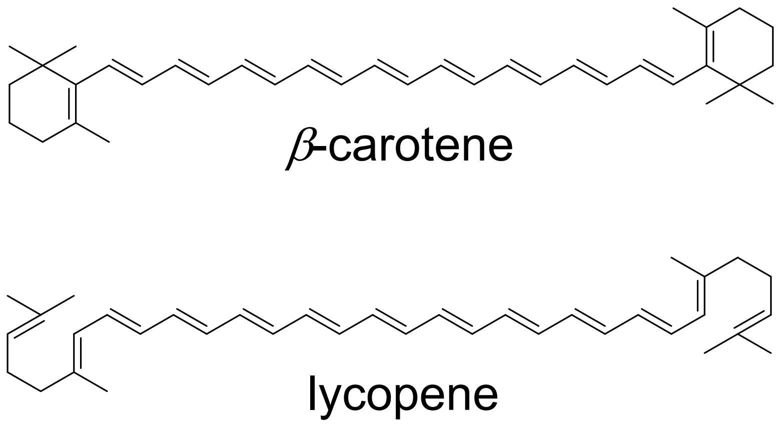

Figure S1. Chemical structure of lycopene and *β*-carotene.

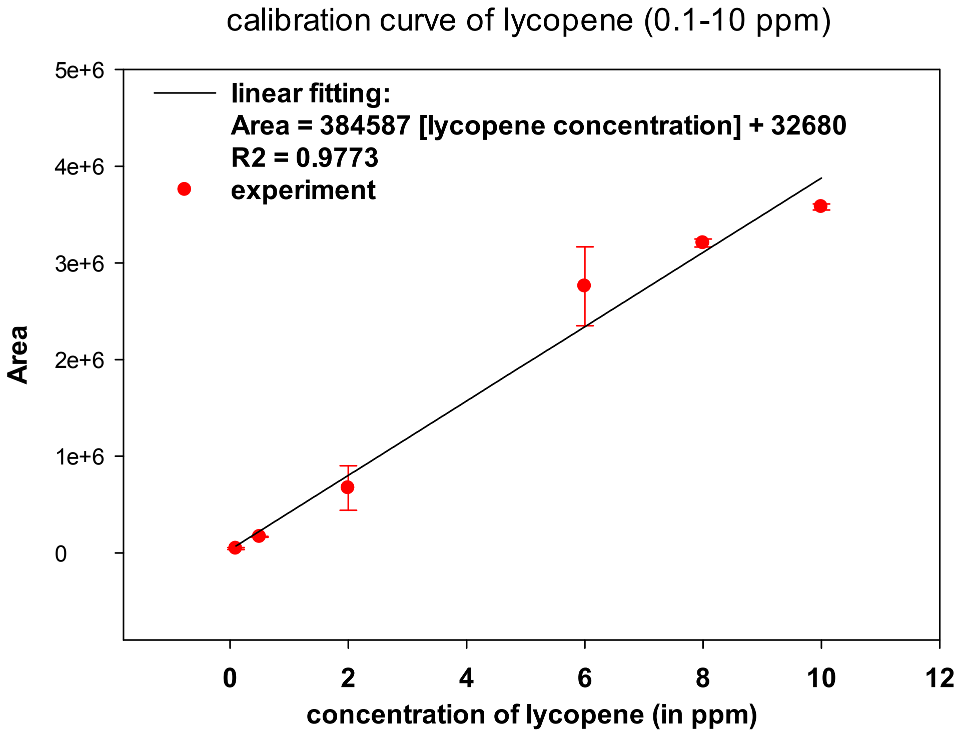

Figure S2. Calibration curve of lycopene in the concentration range of 0.1-10 ppm.

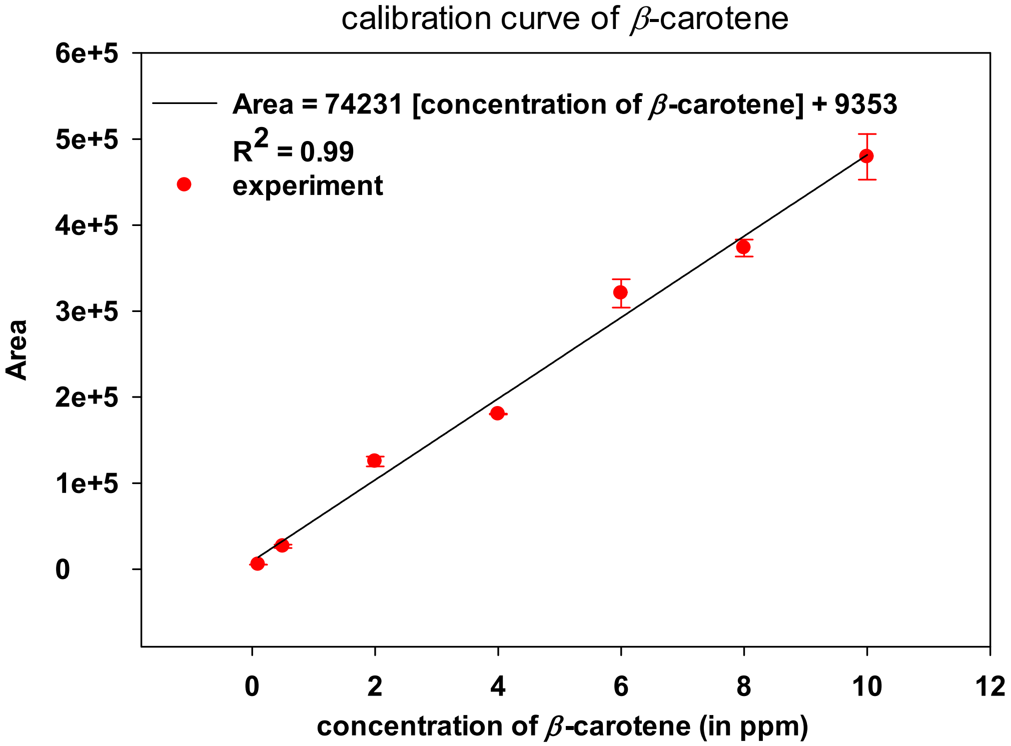

Figure S3. Calibration curve of β-carotene in the concentration range of 0.1-10 ppm.

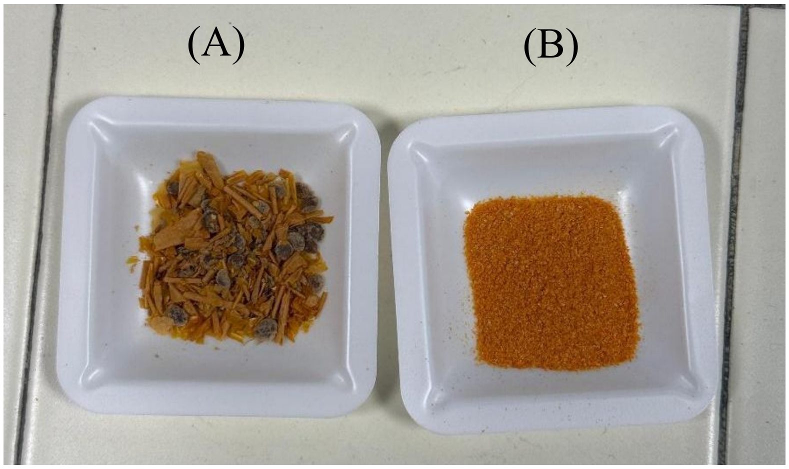

Figure S4. The freeze-dried granules of tomato peels before (A) and after (B) grinding.

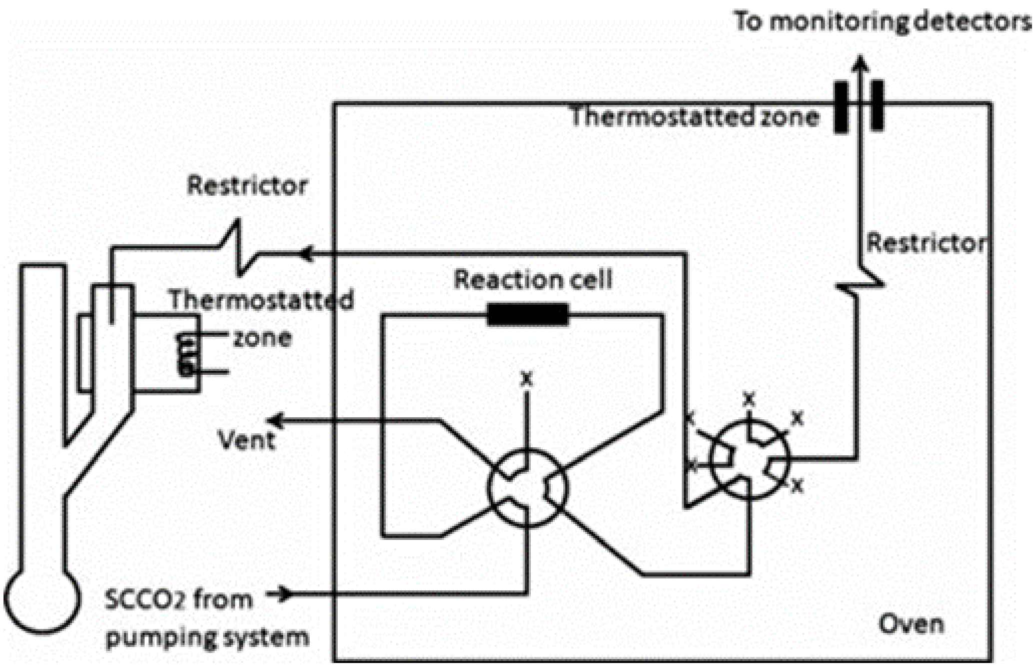

Figure S5. The scheme of the extraction system.

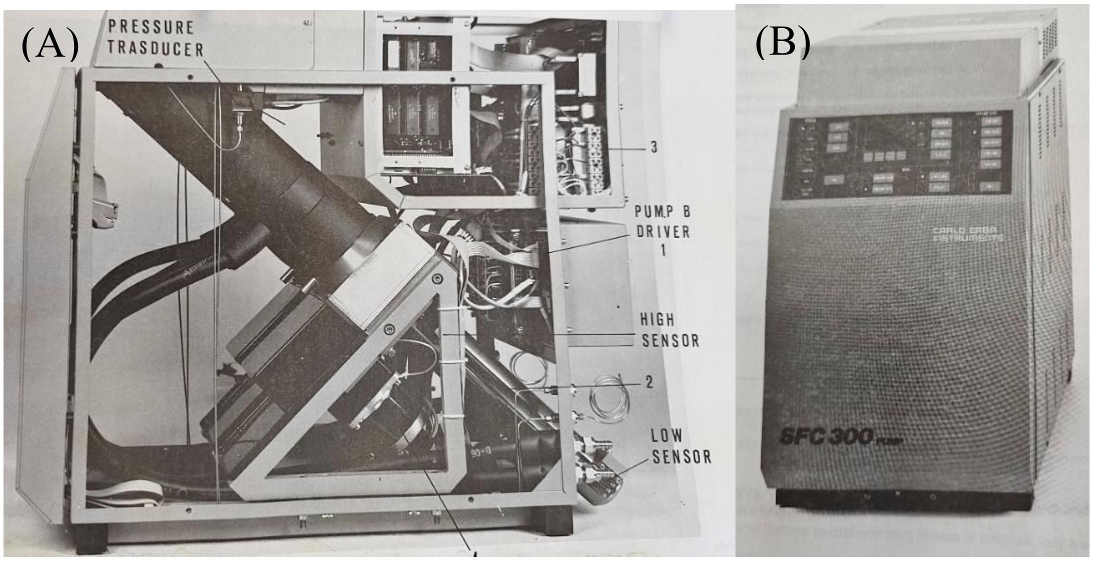

Figure S6. (A) Side view (internal) part of the $scCO_2$ instrument and (B) its processing keyboard.

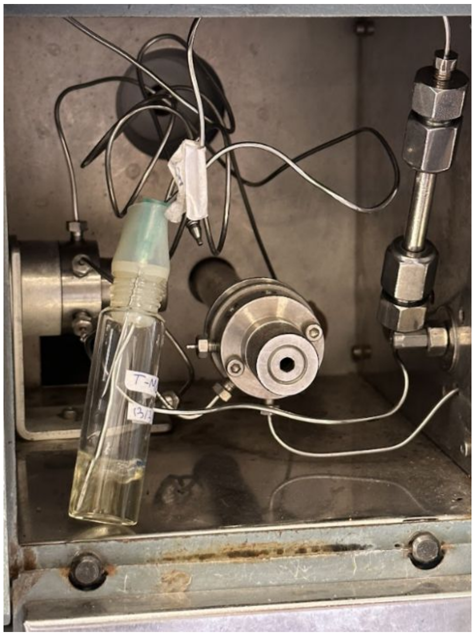

Figure S7. Collecting carotenoids from the cell into 3 mL of ethanol, after the $scCO_2$ extraction.

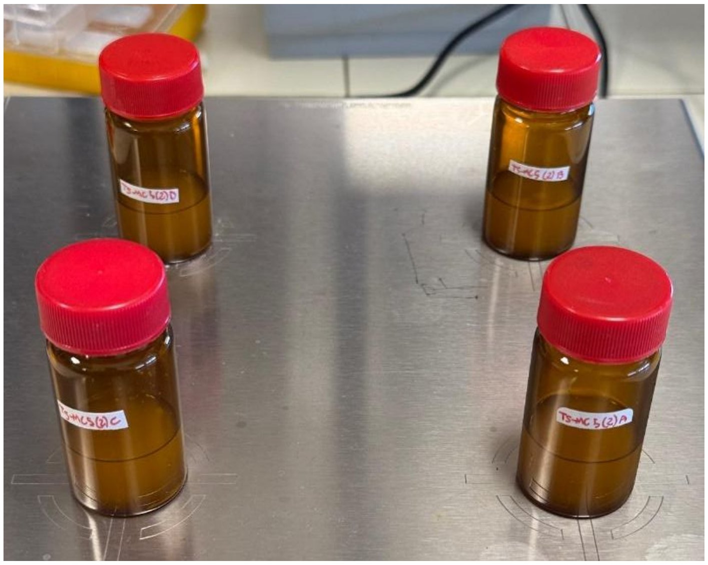

Figure S8. Conjugation of TiO_2NPs with pure lycopene (or β-carotene).

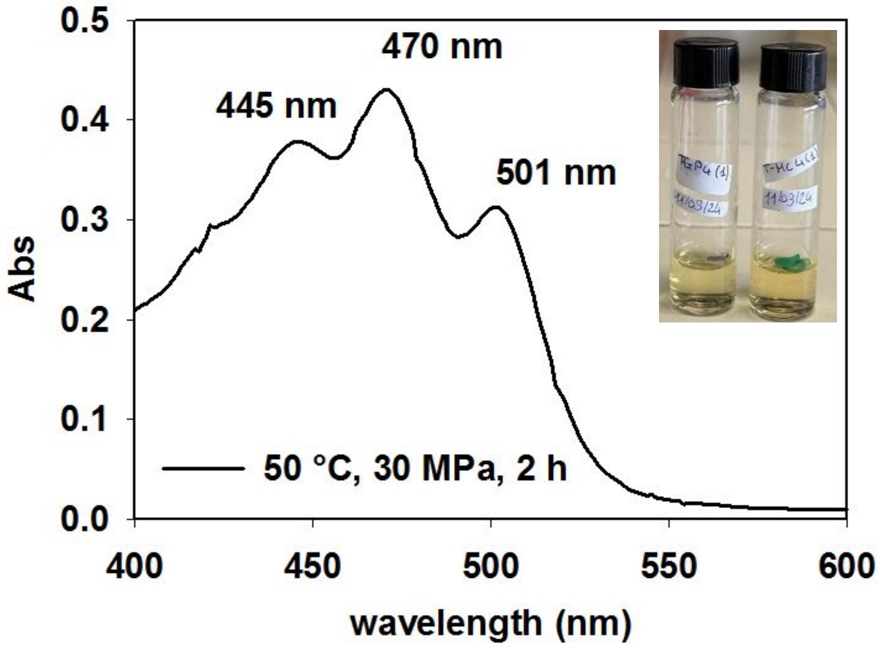

Figure S9. UV-Vis spectrum of the $scCO_2$ extracted sample at 50 °C, 30 MPa, 2 h (inset: the photo of the extracted samples).

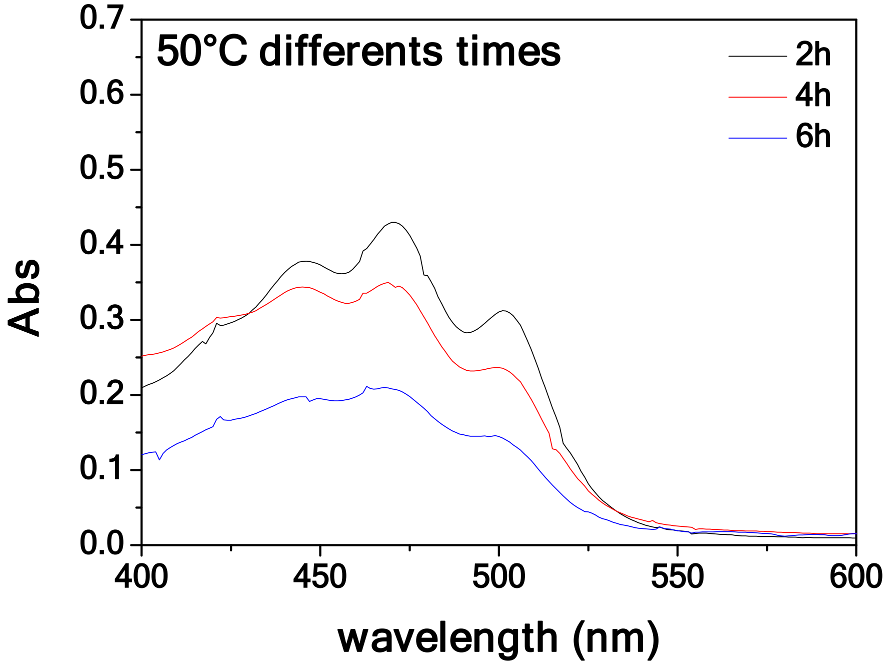

Figure S10 UV-Vis spectrum of $scCO_2$ extracted sample at 50 °C, 30 MPa, for different times.

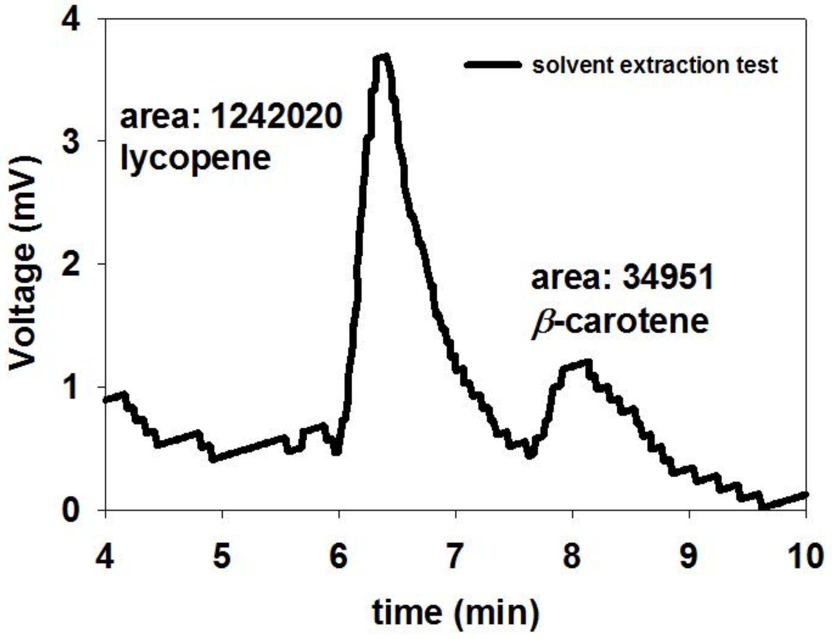

Figure S11. HPLC chromatogram of the solvent extraction test.

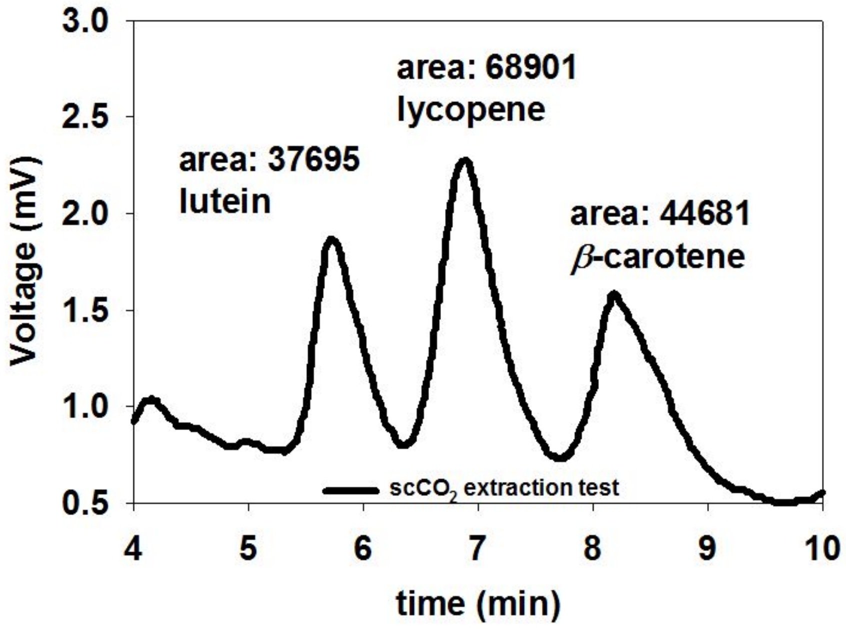

Figure S12. HPLC chromatogram of the best $scCO_2$ extraction test (at 50 °C, 30 MPa, and 2 h).

Table S1. The five selected reaction conditions for the TiO_2NPs-lycopene conjugation.

TiO_2NPs (mg)	reaction volume (mL)	reaction time (hour)	loading%
1	7	3	(3.25±4.3)%
1	7	24	(38.21±6.2)%
1.4	7	24	(95.0±2.1)%
5	7	3	(17.2±3.3)%
5	7	48	(70.15±2.6)%
